# Toxicological effects of copper on bioaccumulation and mRNA expression of antioxidant, immune, and apoptosis-related genes in Chinese striped-necked turtle (*Mauremys sinensis*)

**DOI:** 10.3389/fphys.2023.1296259

**Published:** 2023-11-09

**Authors:** Zeeshan Ali, Ijaz Khan, Muhammad Shahid Iqbal, Qiongyu Zhang, Xiaoqi Ai, Haitao Shi, Li Ding, Meiling Hong

**Affiliations:** Ministry of Education Key Laboratory for Ecology of Tropical Islands, Key Laboratory of Tropical Animal and Plant Ecology of Hainan Province, College of Life Sciences, Hainan Normal University, Haikou, China

**Keywords:** copper toxicity, bioaccumulation, antioxidant and immunity, apoptosis, *Mauremys sinensis*

## Abstract

Heavy metals are among the most ubiquitous environmental pollutants of recent decades. Copper is commonly used to control algal blooms or macrophyte and waste infestations, its ambient concentration has increased significantly, indicating possible environmental risk. To investigate the effects of copper exposure on bioaccumulation, antioxidant defense, immune response, and apoptosis in the Chinese Striped-necked Turtle *Mauremys sinensis,* three experimental groups, control (0.0 mg/L), Cu2 (2 mg/L) and Cu4 (4 mg/L) were designed, and sampled at 14 and 28 days. Results showed that copper accumulates in different organs depending on the concentration and exposure time, Liver > Kidney > Gut > Heart > Brain > Muscle and the time order was 28 days > 14 days. The liver enzymes AST, ALT, and ALP decreased when the turtles were exposed to copper stress, while the contents of bilirubin TBIL, DBIL, IBIL, and LDH showed a significant upward trend. Similarly, the mRNA expression level of acetylcholinesterase AChE in the brain was significantly downregulated upon copper exposure. An upward trend was noticed in the liver Metallothionein MT mRNA expression levels compared to the control group. The mRNA expression levels of antioxidant enzymes CAT, SOD, MnSOD, and GSH-PX1 in the liver increased initially and then significantly decreased. Furthermore, the relative mRNA expression levels of inflammatory cytokines IL-1β, IL-8, TNF-α, and IFN-γ involved in inflammatory response significantly upregulated. Copper significantly increased the hepatic mRNA transcription of heat shock proteins HSP70 and HSP90 at different exposure durations. In addition, the relative mRNA levels of caspase3, caspase8, and caspase9 related to the caspase-dependent apoptotic pathway significantly increased under copper stress. These results explain that copper toxicity causes bioaccumulation, promotes oxidative stress, obstructs immunity, and induces inflammation and apoptosis by altering their gene expression levels in *M. sinensis.*

## 1 Introduction

Copper is one of the essential heavy metals that is not toxic in its metallic form however, some of its salts can be toxic, especially sulfate salts or the blue vitriol (Nila Tutia) and the Verdigris (Zangal), copper sulfate is a crystalline salt having metallic taste with a blue color, a small dose like 0.5 g could be aperient but in higher doses it act as an irritant poison causes bioaccumulation, intestinal and gastric annoyance (Ashish et al., 2013). Biochemical parameters are vital biomarkers in diagnosing the physiological health status of aquatic organisms exposed to various toxicants (Ali et al., 2021). Specific liver enzymes and bilirubin contents in the blood are sensitive measures of liver toxicity and histo-pathological changes that can be assessed within a shorter time (Liu et al., 2010) Due to their capacity to pass the blood-brain barrier, they cause oxidative stress and affect the metabolism of certain proteins implicated in neurodegeneration. Neurochemical changes are also brought on by copper exposure (Richetti et al., 2011). Acetylcholinesterase (AChE) is an enzyme that maintains levels of acetylcholine, an important neurotransmitter that plays a vital role in percipient processes. An acute cholinergic syndrome is the result of acute copper exposure leading to AChE inhibition, which further encourages the accumulation of acetylcholine at synaptic connections and ultimately results in death. (Hsieh et al., 2001). Being a heavy metal copper is commonly known as a critical wheedler in the production of reactive oxygen species ROS such as radicals of superoxide, hydrogen peroxide, and hydroxide which causes oxidative stress and affects the osmoregulatory functions, in addition, it also causes tissue damage (Dautremepuits et al., 2004). Commonly ROS production is balanced by the antioxidant defenses in case of oxidative stress. Superoxide dismutase SOD, manganese superoxide dismutase MnSOD, catalase CAT, and glutathione peroxidase GSH-PX1 are considered indicators of cellular defense mechanisms against ROS and are therefore important tools as biomarkers of pollutant exposure due to the connection between ambient xenobiotic and biological markers of oxidative stress (Morcillo et al., 2015). One of the most important processes taking place in aquatic animals to prevent metal toxicity is detoxification, which is carried out by heavy metal binding proteins called metallothioneins MTs (Achard et al., 2004). The MT concentration of specific organs such as the liver, kidney, and gills rises in aquatic organisms as they withstand metal exposure for a long time which increases the intracellular copper concentrations. To monitor copper contamination in the aquatic environment MT gene expression acts as a reliable biomarker for assessing the toxic effects of copper exposure (Kim and Kang, 2016). Inflammatory cytokines IL-1β, IL-8, TNF-α, and IFN-γ are the indicators of inflammation caused by various environmental stress (Jiao et al., 2019). Similarly, it is thought that HSPs serve crucial roles in defending cells against oxidative stress because they act as molecular chaperones, refolding stress-denatured proteins, inhibiting protein aggregation, or helping in the folding of nascent proteins. (Parsell and Lindquist, 1993). Even though the target protein’s aspartic acid residue can be broken by the cysteine proteolytic enzyme caspase, it cannot break the peptide bond, caspase activity is a valuable diagnostic for detecting stress-induced apoptosis in living animals (Fulda and Debatin, 2006). Many species of vertebrates have yet been reported that act as environmental contamination biomarkers, mainly metal pollution studies (Fujihara et al., 2003).

Freshwater animals, including turtles, play a crucial role in preserving the aquatic ecosystem’s equilibrium and serving as markers of a robust aquatic ecosystem. Turtles are long-lived animals and tend to accumulate higher levels of metals in their different body tissues than observed in water, that is why considered an adequate chemical contamination indicator (de la Lanza-Espino et al., 2000). The Chinese striped-necked turtle (*Mauremys sinensis*) is one of the most common reptile species and is widespread in China, which is known for its economic and pharmacological value (Li-Rong et al., 2015). Various environmental stressors threatened their survival and were included in the IUCN Red List. Heavy metal accumulation and concentration vary among different species and also depend on geographic locations, seasons, tissue types, trophic levels, sex, size, body conditions, and age class (Cortés-Gómez et al., 2017). Copper exposure in common has been linked with organisms’ enzyme inactivation and protein denaturation, leading to detrimental effects like oxidative damage, developmental disorders, neurological damage, initiation of apoptotic pathways, and death (Decataldo et al., 2004). According to earlier research, the use of anti-fouling agents destroys freshwater habitats and interferes with normal metal stability, releasing copper into aquatic ecosystems by direct and indirect routes as in runoff and leaching from agricultural, commercial, and industrial areas. (Vignardi et al., 2023). Although various studies have been conducted on copper toxicity in many freshwater species such as *Cyprinus carpio* (Eyckmans et al., 2011) barnacle larvae (Li et al., 2019) and *Pelodiscus sinensis* (Chen et al., 2019) while the effects of copper on *M. sinensis* have not been studied. The current study investigated copper toxicity and its effect on bioaccumulation, serum liver enzymes, and blood bilirubin levels of *M. sinensis*. In addition, the transcriptional changes of acetylcholinesterase, metallothioneins, and some genes of antioxidant enzymes, innate immune system genes, apoptosis-related genes, and of heat shock proteins in *M. sinensis* were analyzed. This information might aid in the preservation of this threatened species and offer new perspectives on the cellular and molecular causes of copper toxicity.

## 2 Material and methods

### 2.1 Turtles collection and acclimatization

120 healthy *M. sinensis* (51.66 ± 1.55 g) were bought from a local turtle farm and acclimated at room temperature for 1 month in a freshwater tank. The water quality parameters were monitored daily with steady values of pH 7.5–7.9, temperature (26°C–28°C) and the photo period of 12 h throughout the study. During this period, turtles were fed once a week with a commercial diet, after feeding the unconsumed feed was siphoned out by replacement of one-third of the water volume. The contents of the feed were (Protein 38%, fat 4%, ash 16%, fiber 8%, moisture 10%, calcium 4.5%, phosphorus 1.5%, and lysine 1.5%).

### 2.2 Experimentation and sampling

After acclimatization, turtles were divided into three groups, control, Cu2 and Cu4 arbitrarily. 40 juveniles were introduced into each cohort. To make stock solutions, copper was added as CuSO_4_.5H_2_O, which was dissolved in distilled water. The proper volume of the original stock was diluted to form each test solution used in the experiment. The following concentrations were used [0 (control), 2, and 4 mg/L]. The copper concentration was measured twice within 24 h with a water quality analyzer Oakdan®OCT-B Rapid (Octadem, Wuxi, China) with a copper detection range of 0.01–5 mg/L in water. After 14 and 28 days of copper exposure, twelve turtles from each group were randomly collected and anaesthetized according to their body weight with pentobarbital sodium injections. The head were cut off and liver, kidney, intestine, heart, brain, and muscles were then removed. Each turtle received three copies of its organs, except for the heart, kidney, and brain, which were taken as a whole for the determination of bioaccumulation and RNA extraction. We stored all organs at −80°C in liquid nitrogen. For biochemical analysis, 3–5 mL of blood was collected from each turtle from the aorta in the heart without anticoagulant. All experimental methods were authorized by the Hainan Provincial Education Center for Ecology and Environment’s Animal Research Ethics Committee (HNECEE-2014-004).

### 2.3 Bioaccumulation analysis and measurement

100 mg of tissue from the liver, kidney, intestine, heart, brain and muscle were taken from the turtles of each group. All of them were then digested in 10 mL nitric acid for 48 h. The fully digested samples were then boiled on an electric hot plate (Gallen Kamp England) at 100°C in a hood and then cooled to room temperature, each sample was diluted with 30 mL of distilled water and filtered with What-Man filter paper, a purified filtrate was collected in sterilized labeled glass bottles. The liquid filtrate was then analyzed by atomic absorption spectroscopy (Model: Analyst 700, Parkin Elmer, United States, and Serial No: 700S5040102) for the detection of copper.

### 2.4 Serum biochemical analysis

A 15-min centrifugation at 3,000 rpm was used to obtain blood plasma. According to the procedures outlined in (Reitman and Frankel, 1957; Zhao et al., 2020), the levels of alkaline phosphatase, alanine aminotransferase, aspartate aminotransferase, direct bilirubin, indirect bilirubin, total bilirubin, albumin, and lactate dehydrogenase were determined.

### 2.5 Total RNA extraction and qRT-PCR

A 100 mg sample of liver tissue was lysed with TRIzol reagent (Invitrogen United States) and chloroform was used to extract RNA. The quality and purity of mRNA was assessed by measuring their absorbance at 260 and 280 nm using a NanoDrop 2000 spectrophotometer (NanoDrop Technologies, United States), and its integrity was tested by agarose gel electrophoresis. In addition, a prime Script RT-PCR reagent kit from Takara Japan was used to convert the extracted RNA to cDNA. The cDNA templates were then kept at −20°C for further investigation. All primers were designed using the gene sequences found in transcriptome data using NCBI to probe the expression of selected genes (Table 1). Prior to quantifying the target genes, the viability of qRT-PCR machines was confirmed. The reference gene was β-actin as it shows stable expression in the control and exposed groups. The Light cycler 480 system (Roche Diagnostics) and Genious 2x SYBR Green Fast qPCR mix (ABclonal technology China) were used for all qRT-PCR analyses. The gene’s transcriptional levels were normalized to β-actin using the 2^−△△ct^ technique.

**TABLE 1:** Primer pairs sequences used for qRT-PCR.

Gene	Accession	Forward primer(5′ to 3′)	Reverse primer(5′ to 3′)
β-actin	XM_039490283.1	GCACCCTGTGCTGCTTACA	CACAGTGTGGGTGACACCAT
CAT	XM_039533717.1	GGCATTGAACCTAGCCCTGA	GTCCTAAACGGTGTCGGTGA
SOD	XM_039526620.1	GCGTCATCAACTTCGAGCAG	CACCTGCACTGGTACATCCA
Mn-SOD	XM_039532068.1	GGGTCACATCAACCACACCA	AAAGGAGCCAAAGTCACGCT
GSH-PX1	XM_039522759.1	GGCTTGTGCATGTGGGAATG	TGCCCACACTGGAGTTACAC
IL-1β	NM_001317048.1	GAAGACCTCTCTCCGGACCT	CGTCCAAGATGCTGCTCAAG
IL-8	XM_039541818.1	TGTCACTCTGTTTACGCAGC	GTCTGAAGTCTGCTTTGTGCAT
TNF-α	XM_008176809.1	TCCATTCCTCTCCGGCATAC	AGATGGACTCGAACCACACC
IFN-γ	XM_034754901.1	TCCATTCCTCTCCGGCATAC	GCTTTCAGTTAGGCTGTCGTTC
Caspase 3	XM_039540535.1	CCGATCTGGTACTGATGCAGA	TTGCTTCCCTGTACGATCATTG
Caspase 8	XM_039494917.1	ACTGGATCTGAGTGCCCCT	CTGCCCTGTCATACCTTGGC
Caspase 9	XM_039507197.1	TCCGTGATAGACCCCTCCAG	AGGACTTGCTCTTCGTCGTC
HSP70	XM_039486367.1	GAACGAAGAGCAGCAGCAAG	AGCCTTCTTGGCTTGAGGAG
HSP90	XM_005302842.4	CACTACACAGGAGGTCCCTGA	GCCACCCTTGCTTTGTTCTC
AChE	XM_048834021.1	ATGCACCTGCTCTCGCC	CTCCGAGTCGTTGCCCG
MT	XM_039502603.1	GAGCCATGGATCCCCAGAAC	GCATTTGCAGGAGTCAGCAC

### 2.6 Statistical analysis

Spss 23.0 software was used to calculate statistical differences by Means ± S.E. (mean ± standard error). The data were checked for uniformity and normality using the homogeneity variance test. One-way ANOVA and *post hoc* multiple comparisons (Duncan, Tukey’s tests) were used for homogeneous and normally distributed data; otherwise, the nonparametric Kruskal-Wallis H-test was used. A *p*-value of less than 0.05 was regarded as statistically significant.

## 3 Results

### 3.1 Copper accumulation in different organs

**FIGURE 1:**
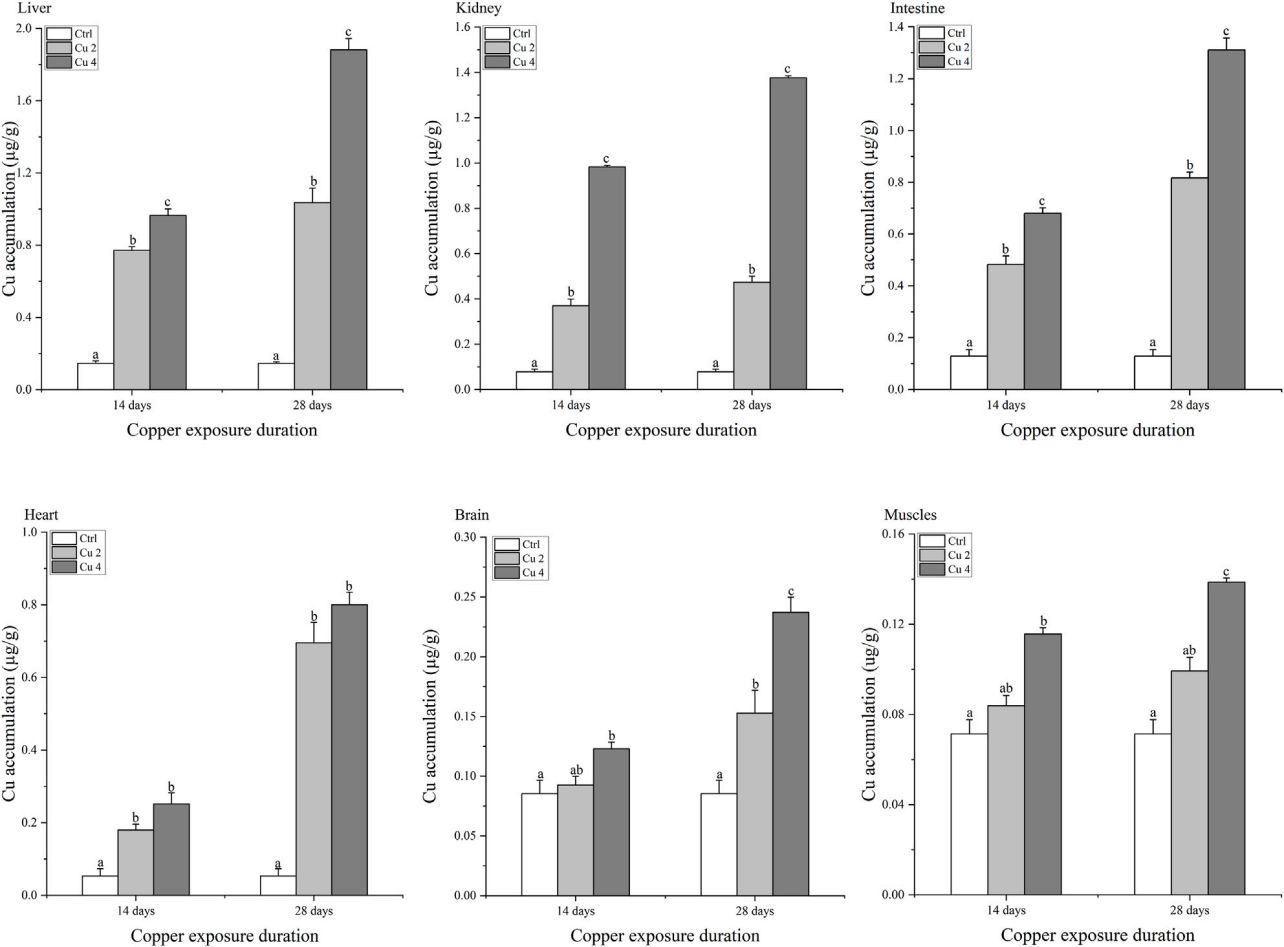
Copper bioaccumulation in the Liver, kidney, intestine, heart, brain, and muscles of *Mauremys sinensis* during copper exposure (*n* = 6). Notes: Ctrl (0.0 mg/L copper concentration), Cu2 (2 mg/L) and Cu4 (4 mg/L). Data are expressed as Mean ± S.E. Bar with different lowercase letters is significantly (*p < 0.05*) different between groups.

Copper accumulation levels in the liver, kidneys, intestines, heart, brain, and muscles of *M. sinensis* during 14 and 28 days are shown in Figure 1. The amount of copper accumulated in various tissues of *M. sinensis* increased over time and at varied concentrations (*p < 0.05*). After 14 days of exposure, the liver showed the highest level of copper bioaccumulation, followed by kidney, intestine, heart, brain and muscle. A similar pattern was observed after 28 days of exposure, but overall accumulation was consistently much higher than at 14 days. The pattern of bioaccumulation compared to organ was liver > kidney > intestine > heart > brain > muscle, while the pattern of bioaccumulation compared to exposure time was 28 days >14 days.

### 3.2 Effect of copper exposure on biochemical parameters

**FIGURE 2:**
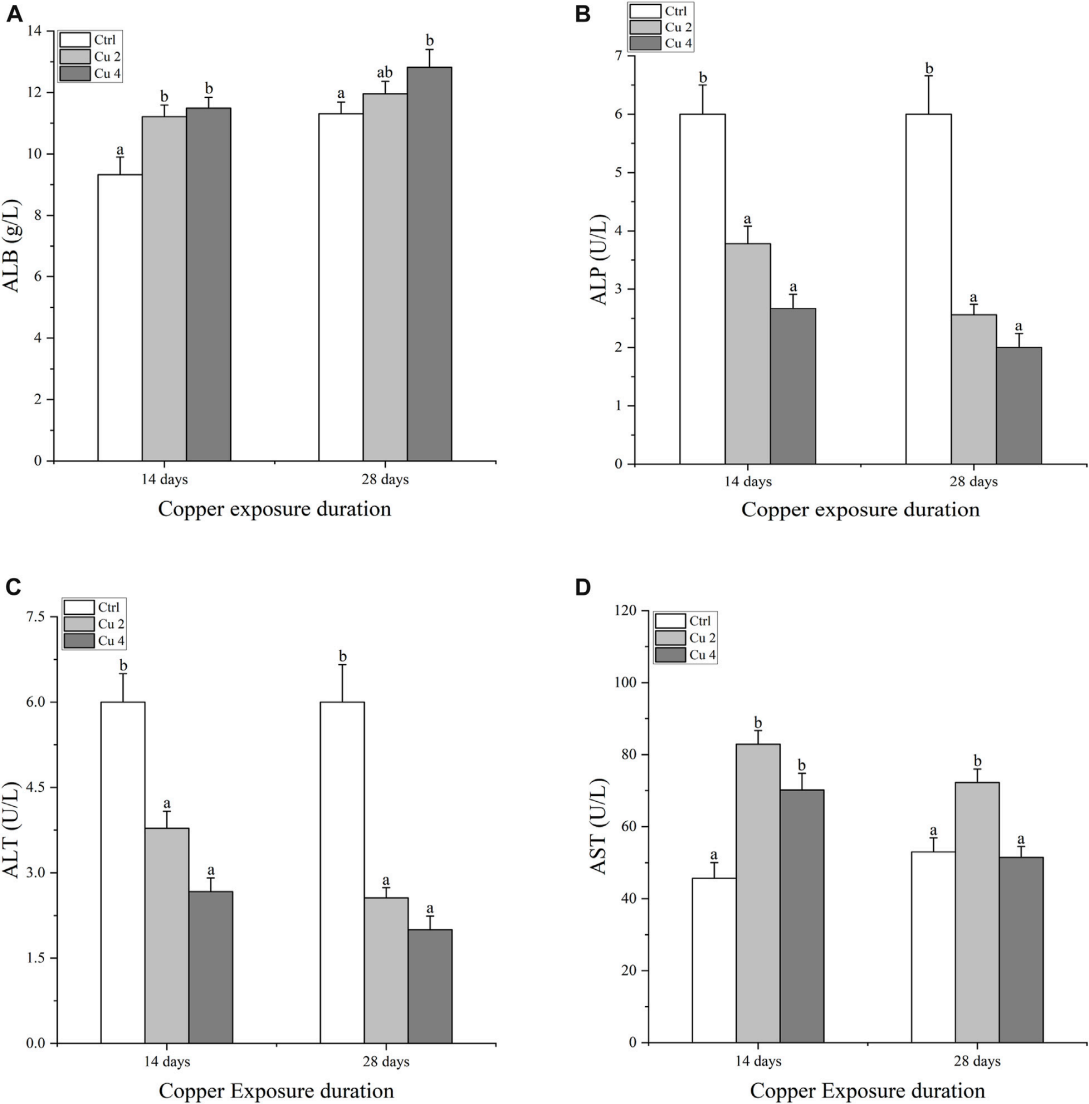
Serum enzyme levels in the blood of *Mauremys sinensis* during copper exposure (*n* = 9) **(A)**: ALB, **(B)**: ALP, **(C)**: ALT, **(D)**: AST. Different lowercase letters **(A–C)** represent the significant difference between groups at the same time (*p < 0.05*).

**FIGURE 3:**
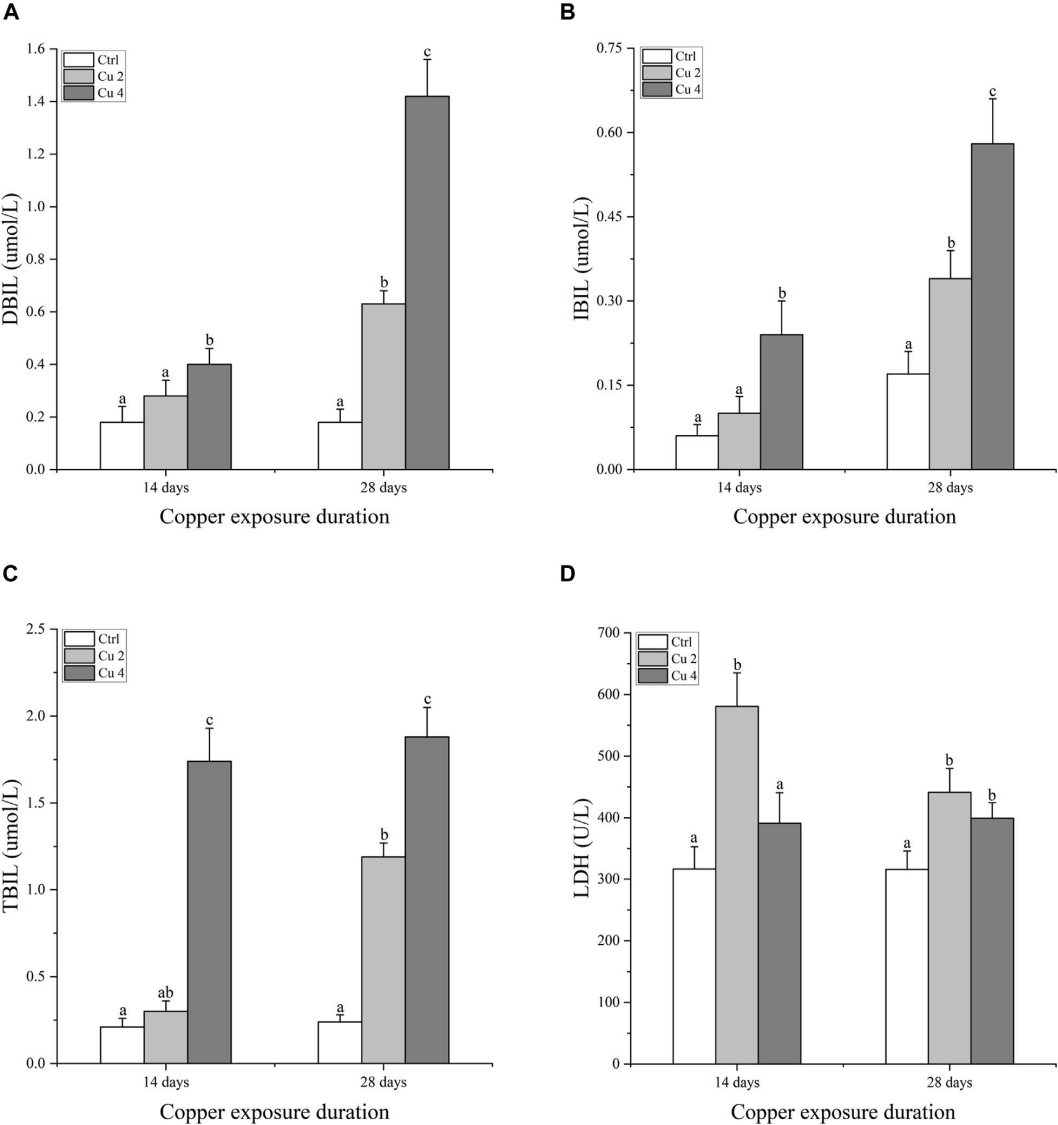
Bilirubin and Lactate dehydrogenase contents in the blood serum of Mauremys sinensis during copper exposure (*n* = 9) **(A)** DBIL, **(B)** IBIL, **(C)** TBIL, **(D)** LDH. Different lowercase letters **(A-C)** represent the significant difference between groups at the same time (p < 0.05).

Serum immune enzymes and proteins are good bio-indicators of monitoring the physiological status of aquatic organisms, compared to reference groups the elevated or inhibited serum enzyme level serves as a diagnostic tool in toxicology and a vital biomarker of metabolic variations in turtles. The present study observed significant changes in serum biochemical parameters, especially in the liver serum enzymes. Serum AST, ALT, ALP, and LDH levels were all affected considerably, AST was significantly increased in both Cu2 and Cu4 groups (*p < 0.05*) as compared to the control group in 14 days of copper exposure, and the same trend was observed in 28 days exposure with decreased values. ALT and ALP levels decreased in both groups and durations as compared to the control. Similarly, serum levels of TBIL, DBIL, and IBIL showed a significant increase (*p < 0.05*) in both treated groups. A slight but signified elevation was noticed in the levels of ALB and LDH in both groups after 14 and 28 days of exposure. Results after 28 days of copper exposure are substantially higher than that of the 14 days in all parameters except ALT, AST, and LDH. The overall outcomes of serum enzymes and bile contents of *M. sinensis* are shown in Figures 2, 3.

### 3.3 Effect of copper exposure on mRNA relative expression of AChE and MT

**FIGURE 4:**
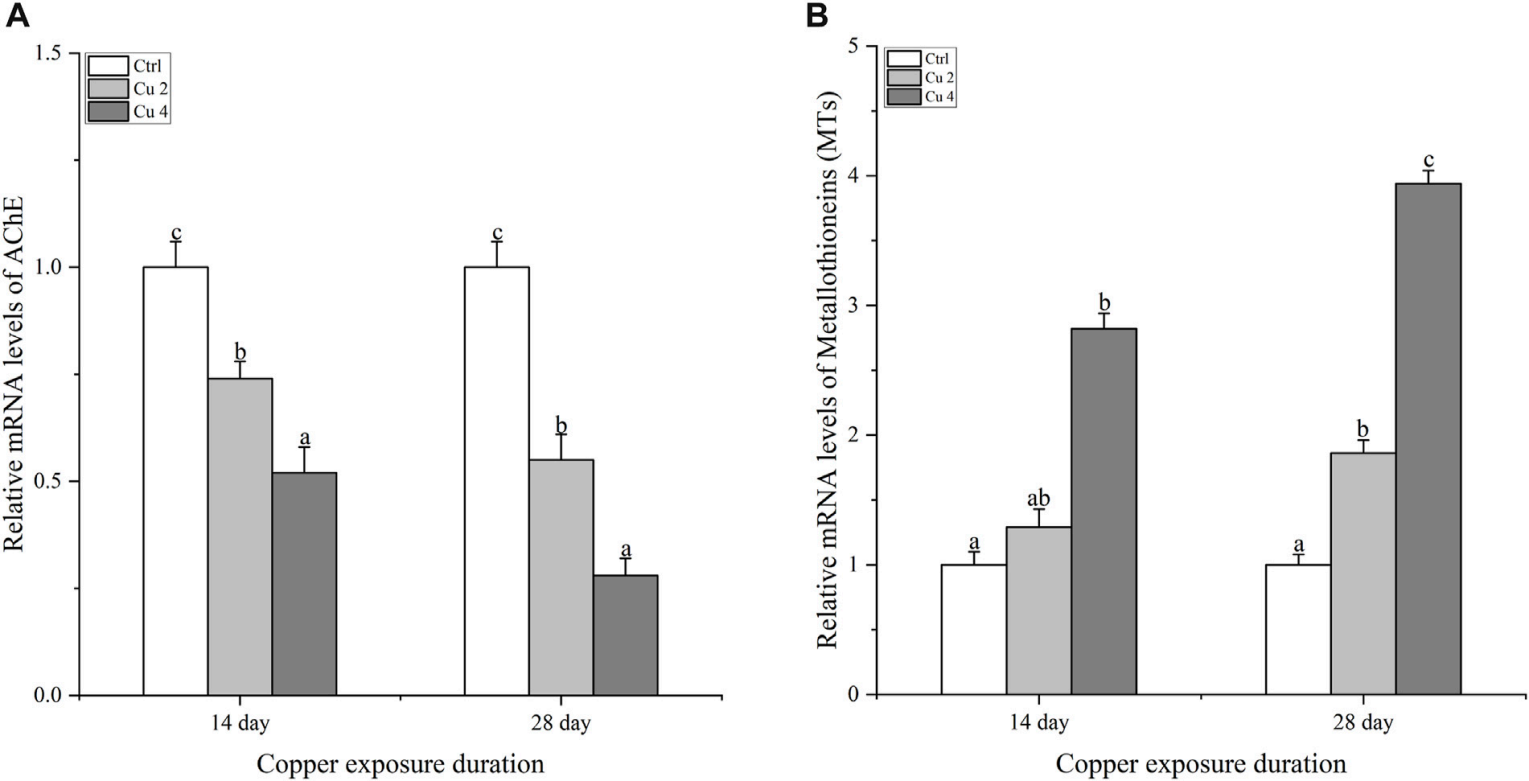
The mRNA relative expression levels of acetylcholinesterase in the brain and metallothioneins in the liver of *M. sinensis* during copper exposure (*n* = 6) **(A)**: AChE, **(B)**: MTs. Bar with different lowercase letters is significantly (*p* < 0.05) different between groups.

Figure 4 shows the gene expression of acetylcholinesterase AChE in the brain of *M. sinensis.* After 14 days of copper exposure, groups Cu2 and Cu4 showed significantly decreased levels of AChE gene expression in the brain. The same significant trend (*p < 0.05*) was also observed in both groups after 28 days of exposure and the lowest level of gene expression was seen in group Cu4. In addition, the level of hepatic metallothioneins MTs gene expression of *M. sinensis* is also shown in Figure 4. Results showed that exposure to water-based copper increased MTs gene expression compared to control. Hepatic MT gene expression showed a slight increase in the Cu2 group on day 14, but it increased significantly in the Cu4 group. At day 28, it showed a significant increase (*p < 0.05*) in both groups and the highest expression was detected in group Cu4.

### 3.4 Effect of copper exposure on the mRNA relative expression of antioxidant enzymes

**FIGURE 5:**
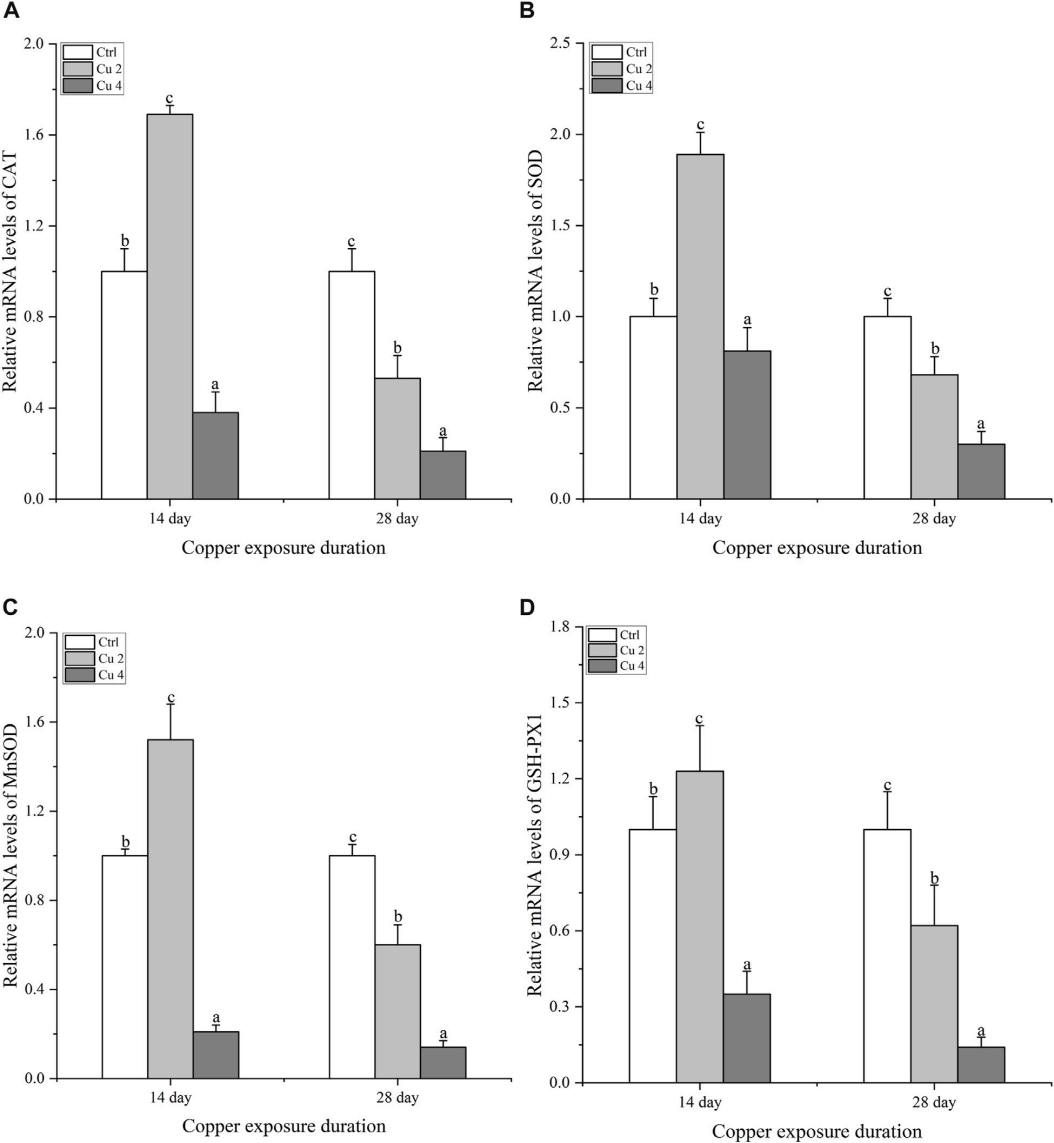
The mRNA relative expression levels of antioxidant enzymes in the liver of *M. sinensis* during copper exposure (*n* = 6) **(A)** CAT, **(B)** SOD, **(C)** MnSOD, **(D)** GSH-PX1. Bar with different lowercase letters is significantly (*p < 0.05*) different between groups.

The relative mRNA expression levels of CAT, SOD, MnSOD, and GSH-PX1 were evaluated to differentiate the expression patterns of antioxidant enzyme genes sensitive to copper exposure (Figure 5). The gene expression levels of hepatic CAT increase slightly in the Cu2 group and decrease in Cu4 after 14 days of exposure compared to the control (Figure 5A). However, after 28 days of exposure, a significant decrease (*p < 0.05*) in hepatic CAT mRNA expression levels was observed in the Cu2 and Cu4 groups. After 28 days, the Cu4 group’s relative CAT mRNA expression level was noticeably lower than that of the Cu2 group. Apart from that, both groups showed a significant decrease in CAT transcription levels by day 28 as shown in (Figure 5A). After 14 days of exposure, the transcription levels of SOD showed an increase and decrease in both treated groups compared to the control (Figure 5B). Similarly, there is a significant increase (*p < 0.05*) in the mRNA transcription level of SOD in the group Cu2, while the transcription level of the group Cu4 decreases significantly by day 28 (Figure 5B). Furthermore, MnSOD transcription level in group Cu2 increases significantly after 14 days and then decreases significantly (*p < 0.05*) at day 28, group Cu2 shows significant increase compared to Cu4 at day 14 of copper exposure (Figure 5C), Even though both groups’ transcription levels had substantially dropped by day 28. Furthermore, the relative mRNA transcription level of GSH-PX1 increased significantly in group Cu2 (*p < 0.05*) and decreased in group Cu4 on day 14 but, decreased significantly (*p < 0.05*) in both the groups on day 28 equated to the control (Figure 5D). The overall results indicated significant changes in hepatic relative mRNA expression levels of the named antioxidant enzymes after exposure to copper stress.

### 3.5 Effect of copper exposure on mRNA relative expression of the innate immune system

**FIGURE 6:**
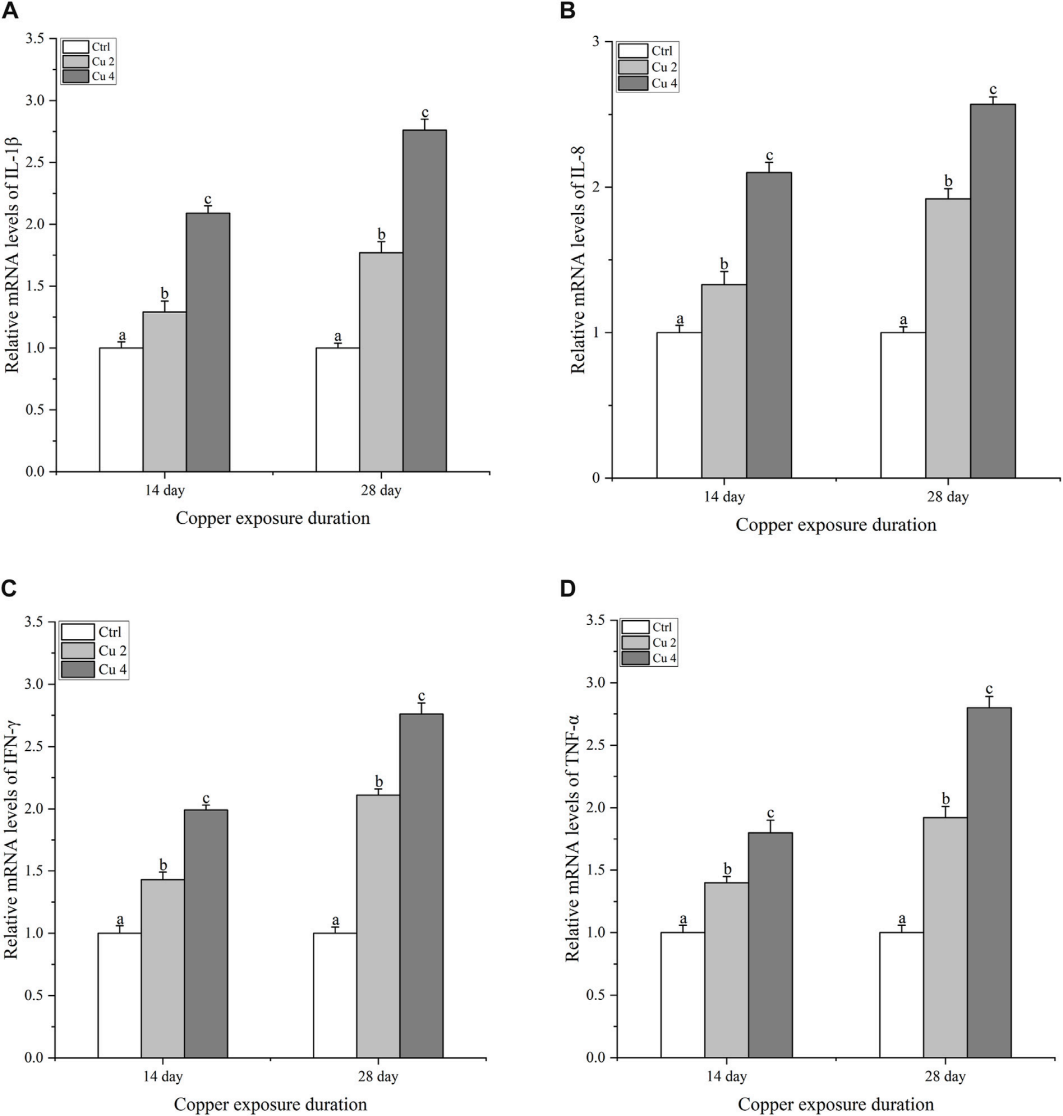
The mRNA relative expression levels of inflammatory cytokines in the liver of *M. sinensis* during copper exposure (*n* = 6). **(A)** IL-1β, **(B)** IL-8, **(C)** IFN-γ, **(D)** TNF-α. Bar with different lowercase letters is significantly (*p < 0.05*) different between groups.

After exposure to copper stress, real-time quantitative PCR was used to determine the amount of innate immune system genes (IL-1β, IL-8, TNF-α, and IFN-γ) transcribed in the liver of *M. sinensis.* Compared to the control group, the transcription levels of all immune genes of both treated groups, Cu2 and Cu4, revealed a significant increase (*p < 0.05*) when exposed to copper for 14 and 28 days and the increase almost doubled at day 28, compared to control as shown in (Figure 6).

### 3.6 Effect of copper exposure on mRNA relative expression of inflammation and apoptosis-related genes

**FIGURE 7:**
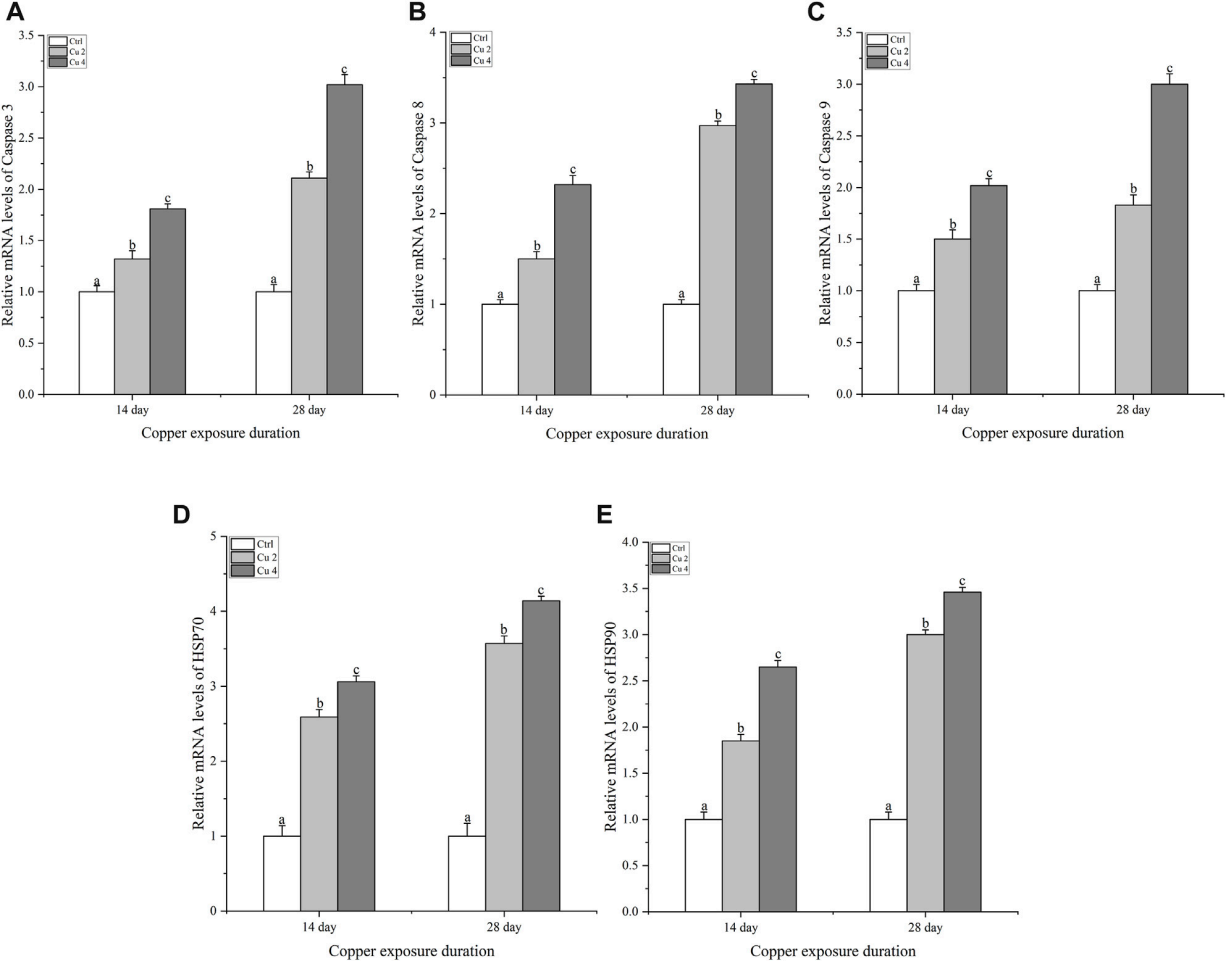
The mRNA relative expression levels of apoptosis-related genes in the liver of *M. sinensis* during copper exposure (*n* = 6) **(A)** Caspase 3, **(B)** Caspase 8, **(C)** Caspase 9, **(D)** HSP 70, **(E)** HSP 90. Bar with different lowercase letters is significantly (*p < 0.05*) different between groups.

To figure out if copper stress triggers apoptosis via the caspase pathway in *M. sinensis*, the transcription levels of caspase 3, 8, 9, HSP70, and HSP90 were examined in the liver. Compared to the control group, there was a significant increase (*p < 0.05*) in caspase 3, 8 and 9 mRNA levels after exposure to two different copper concentrations for 14 and 28 days (Figure 7). At both duration, the expression levels of all three genes were greater in the Cu4 group, while the overall results indicated that the exposure duration and the concentration of copper significantly altered the transcription levels of the genes, which is why caspase 3, 8, 9 bared a significant upsurge (*p < 0.05*) in the transcription levels in group Cu4 at day 28. Similarly, the same trend was also observed in HSP70 and HSP90 and there was a significant upregulation (*p < 0.05*) in their transcription level after copper exposure.

## 4 Discussion

The rapid advancement of industrial technologies is contributing to the rise and worsening of heavy metal pollution. (Yu et al., 2021). According to Chinese Environment Protection Bureau (CEBP) in 1989, the fresh water copper concentration was limited to 0.01 mg/L, Though the concentrations of copper in many copper-polluted freshwater have reached above 560 μg/L natural water, which is much higher than the freshwater-quality standards in many countries, including China (Bu et al., 2022). Copper concentration in aquatic systems has risen in recent years due to industrial effluent discharge, the use of Cu-containing insecticides in agriculture, and mining activities (Lance et al., 2013) which posed a significant health risk to aquatic animals, especially in terms of metabolism and immunity (Yujun et al., 2008). During the past 20 years annual production of Cu in China and the rest of the world has ranked in the top three compared to other metals such as Pb, Cd, Cr, Ni, As and Hg (Fu et al., 2017). This study focuses on the rise of Cu contamination brought on by various anthropogenic activities, and the amount of Cu that pollute canals, streams, rivers, and other freshwater environments daily is increasing, If the concentration rise continues, this must pose a serious threat to the preservation of this endangered freshwater species in the future. Therefore to investigate the physiological parameters and adaptability of *M. sinensis* to copper stress, turtles were exposed to relatively high concentrations of copper.

Juveniles were exposed to different concentrations of copper and copper accumulation was found in six different organs (Liver, kidney, intestine, heart, brain and muscle). The results showed the highest accumulation in the liver. Previous studies have shown that copper accumulates more in the metabolically active tissues of aquatic organisms (Ali et al., 2021). The liver and kidney play a pivotal role in the elimination and revamping of toxic substances therefore its considered to be the target organs for the accumulation of toxic elements (Cortes, 2016). The metabolic activity of the intestine, heart, and brain is ebullient and can easily accumulate toxic substances like, heavy metals (Kim and Kang, 2016). The current study showed that the bioaccumulation of copper in different organs of *M. sinensis* increases with increasing concentration and duration of exposure. The bioaccumulation pattern in the organs of *M. sinensis* is Liver > kidney > intestine > heart > brain > muscles from waterborne copper exposure, This is because when copper enters the body, it first accumulates in the liver, and once it exceeds entry, the excess copper is released into the bloodstream (Liu et al., 2018). For the time being, copper and its toxic compounds enter through ion channels into the cells causing toxicity (Sasso and Schlosser, 2015). There is a difference in bioaccumulation between 14 and 28 days as bioaccumulation increased with increasing exposure time. Small concentrations accumulated in muscles compared to other tissues, since muscles receive the least amount of plasma copper and, unlike other metabolically active organs, are not in direct contact (Sultana et al., 2021). Therefore, a significant increase in muscle bioaccumulation was also noted, but not as significant as in other underlined tissues.

The liver functions as a vital organ for immunity and the breakdown of toxic substances. Liver damage can be easily assessed by the impaired activity of AST, ALT, and ALP. These are important markers of hepatocyte damage (Yu et al., 2021). The hypothalamic-pituitary-adrenal axis (HPA) controls LDH, which is largely found in the liver. It is a crucial enzyme that helps balance endocrine processes and acts as a sensitive indicator of the body’s ability to cope with stress (Kong et al., 2021). An increase in waterborne copper significantly decreases AST, ALT, and ALP activities (Wang et al., 2019). In addition, significant variations in the plasma levels of SGPT and SGOT were observed after exposing common carp to Mn and Cr (Ali et al., 2021). Our current results are consistent with the above research, which further shows that copper exposure causes significant liver damage in *M. sinensis* and results in significant fluctuations in blood levels of AST, ALT, ALP, and LDH. In addition, bilirubin is regarded as a member of the antioxidant family and has harmful effects when its blood levels are high. (Tomaro and del C Batlle, 2002). Similarly, TBIL, IBIL, and DBIL possess potent antioxidant activity against peroxide radicals and protect lipid membranes from peroxidation by acting synergically with vitamin E (Stocker et al., 1990). In our current study, we come to the same conclusions as discussed, and the levels of TBIL, IBIL, and DBIL were significantly increased compared to the control group in *M. sinensis* after exposure to copper stress. Our findings indicated that bilirubin contents increased in the blood as a defensive act against copper toxicity. Acetylcholinesterase AChE maintains acetylcholine ACh levels, which activate various important receptors in the nervous and muscular systems. They do this by catalyzing various reactions involved in this process to maintain acetylcholine ACh levels (Richetti et al., 2011). Previous studies suggested that exposure to copper induces neurochemical changes as it crosses the blood-brain barrier, leading to oxidative stress and causing fluctuations in protein metabolism, particularly implicated in neurodegeneration (Attig et al., 2010). Similarly, a significant decrease in AChE was detected by (Ciacci et al., 2012; Kim et al., 2017) in the brain and muscles of *S. schlegelii* and *Mytilus galloprovincialis* caused by high levels of Cr exposure. The same results were also found in our experiment after exposure to copper stress, there was a significant downregulation of AChE mRNA levels in the *M. sinensis* brain. In addition, metallothioneins are proteins that have the property of detoxifying heavy metals, stabilizing the homeostasis of essential metals including copper and zinc, and providing protection against oxidative stress and apoptosis (Xie et al., 2004). To reduce the problems caused by metal exposure, organisms have created metallothioneins (MTs) as a first line of defense against metal toxicity. Therefore, metallothioneins are a clever and reliable component of aquatic metal defenses against metal toxicity (Andreani et al., 2008). Our results showed a significant increase in the transcription of hepatic metallothioneins in *M. sinensis* after copper exposure, the same results were observed by (Bakiu et al., 2022), a significant MTs gene transcription inducted in the liver of *Trematomus Hanson* against copper stress. This illustrates the antioxidant effect of metallothioneins.

Excessive accumulation of copper in various organs alters antioxidant defense mechanisms and leads to oxidative damage (Chen et al., 2021). Under physiological and environmental stress, turtles and other ectotherms may produce more ROS altering gene expression, leading to per-oxidation of proteins and lipids, and changing the redox state of cells (Sevcikova, 2011). When exposed to copper, freshwater turtles must control the possibility of oxidative stress while promoting physiological responses to account for the change in ambient osmotic pressure. (Abele and Puntarulo, 2004). Additionally, sufficient antioxidant defenses at all times could rapidly stop or reduce the formation or buildup of ROS and hence reduce oxidative damage. (Krivoruchko and Storey, 2010). Superoxide dismutase (SOD), manganese superoxide dismutase (MnSOD), glutathione peroxidase (GSH-PX1), and catalase (CAT) are one of the key antioxidant enzymes that can eliminate excess ROS to reduce damage (Xing et al., 2008). The SOD and its isoform MnSOD catalyze superoxide anion to hydrogen peroxide (Zelko et al., 2002). The CAT converts hydrogen peroxide into water (Asagba et al., 2008), and glutathione peroxidases such as GSH-PX1 can work with SOD to detoxify other peroxides, like lipid peroxides created by free radical impact on membranes or other lipids. (Smith et al., 1999). In the current research, *M. sinensis* was found to have lower gene expression of CAT, SOD, MnSOD, and GSH-PX1 in the liver in response to water-borne copper stress. Similarly, the mRNA expression levels of the underlined enzymes showed upregulation in the group Cu2 at the initial 14-day exposure as an early response to copper stress, but decreased in both the groups after 28 days of copper exposure (Figure 5). Excessive metal levels lead to decrease in CAT, SOD, MnSOD, and GSH-PX1 mRNA expression levels and cause oxidative damage. The same results were also found in the research work of (Cortes, 2016). The results suggest that turtles employed the antioxidant system against increasing ROS, but as the concentration and duration of copper exceeded, the turtles’ response becomes weaker. The same results were observed in the study by (Yu et al., 2021)), in which the antioxidant enzymes gene expression pattern decreased significantly by exposing *Channa asiatica* to hexavalent chromium (Cr^6+^) for 28 and 56 days. Similarly, our results are also consistent with the results of (Cortés-Gómez et al., 2018) where *Lepidochelys olivacea* showed marked variations in mRNA levels of antioxidant enzymes.

An excessive amount of ROS generation not only stimulates the body’s antioxidant defense system but also causes an inflammatory reaction (Liang et al., 2020). Many studies have described that copper exposure triggers inflammation and several inflammatory cytokines are involved in the regulation of inflammatory and immune responses (Zhao et al., 2020). Variations in the gene expression of the inflammatory cytokines IL-1β, IL-8, TNF-α, and IFN-γ perceive the inflammatory response. Studies have shown that inflammatory cytokines play an important role in innate immunity by protecting aquatic animals under stressful conditions. In this study, the mRNA of all selected four IL-1β, IL-8, TNF-α, and IFN-γ cytokines were upregulated when *M. sinensis* was exposed to copper stress. The same as arsenic exposure upregulated expression of inflammatory cytokines in *C. carpio* (Wang et al., 2019). This implies that turtles’ immunological and inflammatory systems could be affected by copper exposure. Similarly, the heat shock proteins HSP70 and HSP90 also show upregulation in cells under environmental stress and participate in signaling to minimize damage HSP70 synthesis occurs when the cell’s redox state is disrupted, while HSP90 is highly conserved and expressed in all eukaryotic cells (B et al., 2009). The heat shock response is a highly conserved mechanism. Their job is to protect cells, maintain metabolic status, and improve survival under various environmental stresses (Bienz and Mariann, 1985). Our study also showed that copper stress increased transcription of the HSP70 and HSP90 genes in *M. sinensis* liver. The results are similar to the changes seen in HSP70 and HSP90 of *Channa argus* and *C. carpio* after copper exposure (Wang et al.; Li et al., 2019). According to the findings, heat shock protein is essential for minimizing cellular harm and enhancing the turtles’ resistance to copper stress.

Apoptosis is a programmed cell death and can be triggered by various environmental stressors such as salinity, heavy metals, pH and temperature (Holmgren, 1995). Stress-induced apoptosis can be easily detected by caspase activity. The extrinsic and intrinsic pathways are the two main mechanisms by which cells undergo apoptosis. The extrinsic death receptor pathway involves immediate activation of the initiator caspase8, regulated by identifying extracellular ligands with trans-membrane receptors (Chávez and Gallardo, 2014). Cytochrome c is released from the mitochondria to begin the intrinsic route, which subsequently activates caspase 9 and caspase 3. Caspase 9 and 3 cause a variety of proteins, including fodrin and nuclear laminae, to be cleaved, which eventually causes cell death. (Liu et al., 2013). Our results describe that the gene expression of caspase9, caspase8, and caspase3 in the liver of *M. sinensis* was significantly upregulated after exposure to copper stress. The results were similar to *C. asiatica* when exposed to hexavalent Cr, the caspase 3, 8, and 9 genes showed a significant upregulation (Yu et al., 2021). Likewise, salinity and selenium Se exposure pointedly amplified the mRNA levels of apoptotic genes in *T.s elegans* and *C. argus* respectively (Ding et al., ; Li et al., 2019). The above results are similar to the results of the current experiment and suggest that copper stress in water can lead to Toxicity, bioaccumulation, and causes transcriptional changes in different antioxidant, immune and apoptosis-related genes in Chinese striped-necked turtle (*M. sinensis*).

## 5 Conclusion

According to our findings, copper toxicity and bioaccumulation were seen in *M. sinensis*. The effects of copper exposure on serum liver enzyme levels and blood bilirubin contents point to copper’s potential impact on healthy liver function. The turtles’ antioxidant defense mechanism was also turned on during exposure to help them fight off the harm. However, only in the initial phases of exposure this phenomena occur. The antioxidant system, innate immune system, and heat shock protein genes expression levels were also increased in an effort to protect cells from oxidative stress and death. A persistent copper stress also causes the caspase-dependent apoptotic pathway to be activated, which results in apoptosis. Thus, we have demonstrated that copper stress in the Chinese striped-neck turtle *M. sinensis* results in toxicity, bioaccumulation, and transcriptional changes in many antioxidant, immunological, and apoptosis-related genes. The study advances our knowledge of aquatic creatures’ tolerance levels for stressful situations as well as their living environments.

## Data Availability

The original contributions presented in the study are included in the article/Supplementary materials, further inquiries can be directed to the corresponding authors.
